# Promoter-proximal nucleosomes attenuate RNA polymerase II transcription through TFIID

**DOI:** 10.1016/j.jbc.2023.104928

**Published:** 2023-06-15

**Authors:** Michael J. Fisher, Donal S. Luse

**Affiliations:** Department of Cardiovascular and Metabolic Sciences, Lerner Research Institute, Cleveland Clinic, Cleveland, Ohio, USA

**Keywords:** RNA polymerase II, promoter, nucleosome, TFIID, histone methylation

## Abstract

A nucleosome is typically positioned with its proximal edge (NPE) ∼50 bp downstream from the transcription start site of metazoan RNA polymerase II promoters. This +1 nucleosome has distinctive characteristics, including the presence of variant histone types and trimethylation of histone H3 at lysine 4. To address the role of these features in transcription complex assembly, we generated templates with four different promoters and nucleosomes located at a variety of downstream positions, which were transcribed *in vitro* using HeLa nuclear extracts. Two promoters lacked TATA elements, but all supported strong initiation from a single transcription start site. In contrast to results with minimal *in vitro* systems based on the TATA-binding protein (TBP), TATA promoter templates with a +51 NPE were transcriptionally inhibited in extracts; activity continuously increased as the nucleosome was moved downstream to +100. Inhibition was much more pronounced for the TATA-less promoters: +51 NPE templates were inactive, and substantial activity was only seen with the +100 NPE templates. Substituting the histone variants H2A.Z, H3.3, or both did not eliminate the inhibition. However, addition of excess TBP restored activity on nucleosomal templates with TATA promoters, even with an NPE at +20. Remarkably, nucleosomal templates with histone H3 trimethylated at lysine 4 are active with an NPE at +51 for both TATA and TATA-less promoters. Our results strongly suggest that the +1 nucleosome interferes with promoter recognition by TFIID. This inhibition can be overcome with TBP alone at TATA promoters or through positive interactions with histone modifications and TFIID.

Metazoan RNA polymerase II (pol II) promoters may be divided into two broad categories: a minority that contains a TATA element roughly 30 bp upstream of the transcription start site (TSS) and the large majority that lack that sequence motif (1, 2). The pol II preinitation complex (PIC) makes template contacts well downstream of the TSS, typically extending to about +35 (3, 4, 5). However, except for a minimal initiator element (Inr) around the TSS itself (6, 7), common sequence motifs downstream of the TSS have proven difficult to identify for TATA-less promoters. Downstream elements with various designations have been mapped from ∼+17 to +35, but these motifs are not universally present in promoters (8, 9). Many pol II promoter regions do not direct initiation from a single TSS but instead support TSSs over a wide range of locations (1, 10). In spite of the apparent diversity in sequence motifs and TSS spacing, *in vitro* studies have identified a universal set of core general transcription factors for pol II promoters (reviewed in Refs. (11, 12, 13)). Recent structural studies show that these core factors direct PIC formation at both TATA and TATA-less promoters. The fully assembled PICs of both promoter types occupy essentially the same segment of template DNA relative to the TSS (4). Consistent with this point, *in vivo* mapping of pol II TSSs with nuclear run-on approaches revealed a common sequence signature for all human promoters (9), which extends over the same region bound by the core factor TFIID on promoter DNA *in vitro* (14, 15). Nuclease protection studies in isolated nuclei revealed that this same TFIID footprint is protected by pol II PICs *in vivo* for both TATA and TATA-less promoters (5). However, structural studies also showed that the assembly pathway to the final PIC is different for TATA and TATA-less promoters. This reflects the different sequence biases for initial promoter recognition in the two classes: upstream at TATA for TATA promoters and downstream of the TSS for TATA-less promoters (4, 15, 16).

Along with substantial recent progress in mapping out PIC assembly with pure components, it is increasingly evident that a complete description of the pol II promoter must include the immediate chromatin environment in which the PIC is assembled. Metazoan promoters have a downstream (+1) nucleosome whose proximal edge usually lies just beyond the expected boundary of a PIC (9, 17). Mapping of TSSs by run-on methods in nuclei identified a periodic sequence element extending from 50 to 200 bp downstream of the TSSs, which could serve to position +1 nucleosomes in this location (9). When nuclease-treated nuclei were immunoprecipitated to recover PICs, a significant fraction of the PIC-bearing fragments was much larger than expected for the PIC alone. These DNAs extended to just beyond the expected downstream edge of the +1 nucleosome, indicating that some PICs were directly abutted to the +1 nucleosome (5). All these findings point to the possibility that the +1 nucleosome might have an important positive role in modulating PIC assembly, instead of simply providing a barrier to transcript elongation. A clear mechanistic basis for such a role is the ubiquitous presence in promoter-proximal nucleosomes of histone H3 trimethylated on lysine 4 (H3K4me3; (18, 19)), since this modified H3 is known to interact specifically with the TAF3 subunit of TFIID (20). In addition to histone modifications, the histone composition of the +1 nucleosome is distinctive. Promoter-proximal nucleosomes are enriched in variant histone types, particularly H2A.Z for H2A and H3.3 for H3.1 (21). These substitutions from the canonical histones might also affect PIC assembly, particularly since *in vitro* studies showed that incorporation of these histones results in reduced nucleosome stability (22, 23).

In the experiments reported here, we utilized a nuclear extract–based *in vitro* transcription system to determine the functional importance of promoter-nucleosome distances and nucleosome composition for PIC assembly. We tested mononucleosomal templates with TATA or TATA-less promoters located at a series of distances upstream of a precisely positioned +1 nucleosome. The nucleosomes had variable composition, with some containing H2A.Z, H3.3, or H3K4me3. We show that the +1 nucleosome negatively regulates transcription complex assembly by default, an effect that is much more pronounced on TATA-less promoters. Bypassing TFIID through TATA-binding protein (TBP) supplementation can overcome this inhibition but only on TATA-containing promoters. Crucially, substitution of H3K4me3 for nonmodified H3 overcomes the inhibition for both promoter classes. Our results implicate TFIID as a key regulator in modulating assembly of pol II PIC in a chromatin environment similar to that seen in the nucleus.

## Results

**Figure 1:**
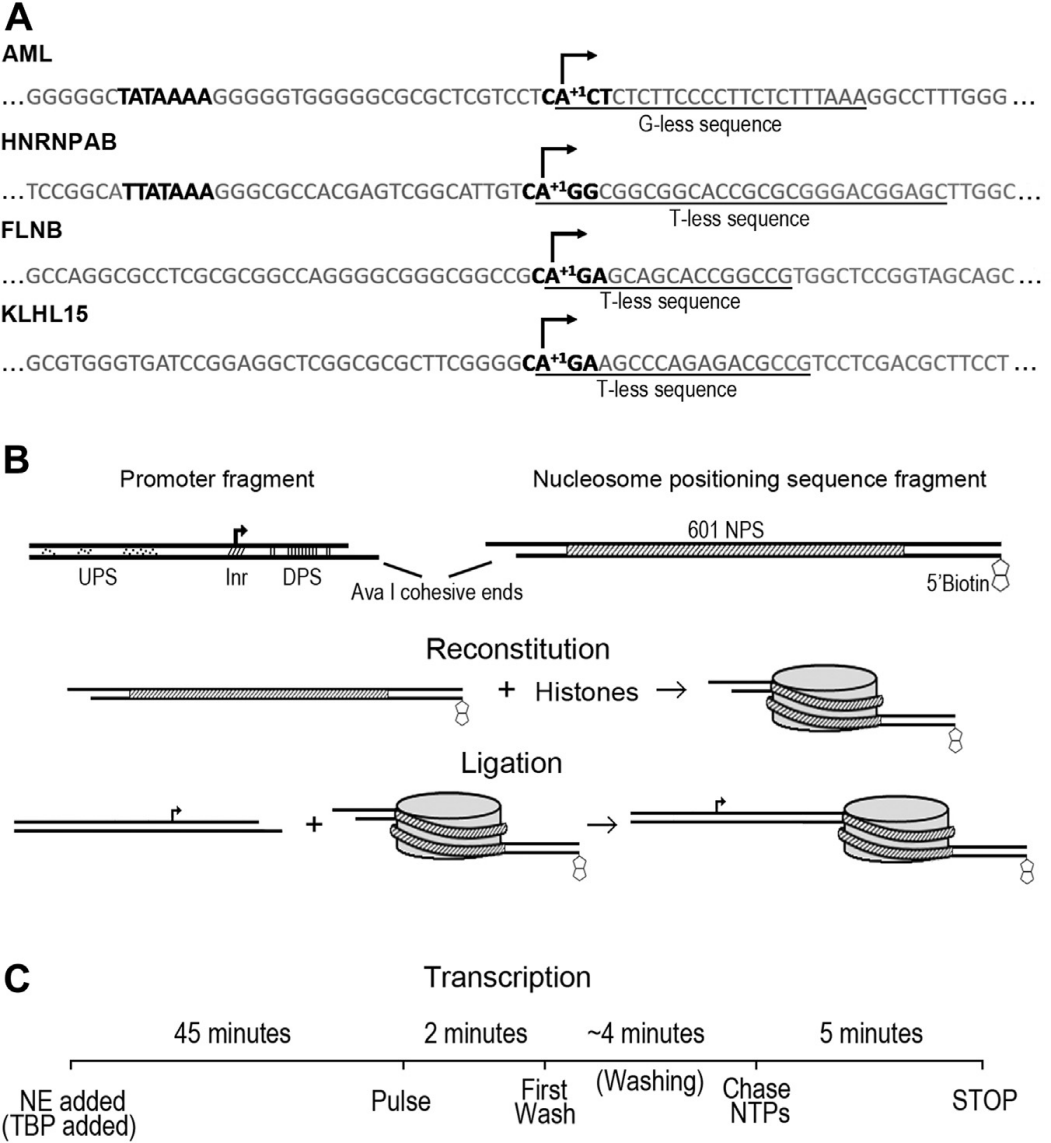
**Sequences and assembly of nucleosomal templates and transcription complexes.***A*, core promoter region of the four templates, with TATA elements (when present), initiator sequences (Inr), and the extent of G or T-free downstream regions indicated. *B*, templates for transcription were ultimately assembled from DNA fragments with upstream (UPS) and downstream (DPS) promoter sequences joined through AvaI sites with varying spacing to biotinylated nucleosomal fragments containing a modified 601 nucleosome positioning sequence (NPS). Nucleosomes were reconstituted on the NPS-bearing fragments, ligated to promoter fragments, and then bound to magnetic beads. *C*, preinitiation complexes were assembled on the bead-bound templates by incubation with nuclear extract for 45 min. Transcription was initiated by adding limiting NTPs and radioactive CTP, incubating for 2 min. The reactions were washed for 4 min ± 30 s, thereby removing unbound proteins and NTPs. Reactions were chased by adding all four NTPs and incubating for 5 min before stopping the reactions.

We wished to investigate the effects of promoter-proximal nucleosomes on pol II transcription through *in vitro* approaches. Our experimental design was intended to encompass not only the range of locations downstream of TSS typical for +1 nucleosomes but also different histone types and modifications frequently associated with the initial downstream nucleosome. Particularly in light of potential mechanistic differences in the pathways of PIC assembly for TATA-containing and TATA-less promoters (4, 15), we included examples of both promoter classes in our analysis. To identify appropriate promoters, we examined TSSs mapped by nuclear run-on in HeLa cells (9). Among the highly expressed promoters, we searched for TATA-containing and TATA-less examples that support single TSSs *in vivo*. To facilitate pulse labeling of transcripts with CTP, we further screened for promoters where the nontemplate strand in the initially transcribed region is C-rich but lacks either G or T (Fig. 1*A*). We selected HNRNPAB as a representative TATA-containing promoter. We also included in the TATA class the core section of well-studied adenovirus major late (AML) promoter, which supports a single TSS and has an engineered G-less cassette downstream (Fig. 1*A*). Among the TATA-less promoters, we selected FLNB and KLHL15. It is worth noting that both TATA-less sequences have consensus initiator elements (6) (Fig. 1*A*). Our template constructs retain the native sequences for at least 96 bp upstream of the TSSs for HNRNPAB, FLNB, and KLHL15.

The complete templates for these assays were assembled by ligation of promoter fragments to downstream DNA bearing a single nucleosome (Fig. 1*B*) (24). Nucleosomes were rapidly reconstituted (25) on a modified 601 positioning element (26) biotinylated at its downstream end. After confirming complete assembly, the nucleosomes were ligated to promoters with variable lengths of sequence downstream of the TSS. We will refer to the distance from the TSS to the proximal nucleosome boundary as the nucleosome proximal edge (NPE) and designate templates by that value. Because of requirements for the ligation, all templates retain a common 16 bp sequence upstream from the NPE. For example, 51 templates with HNRNPAB, FLNB, and KLHL15 retain 35 bp of their native downstream promoter sequence and 100 templates retain 84 bp of native sequence. The ligated templates were conjugated to ferromagnetic beads for convenient manipulation. The transcription assay for nucleosomal and control naked DNA templates is summarized in Figure 1*C*. To successfully support preinitiation complex assembly on TATA-less as well as TATA templates, it was necessary to use nuclear extracts instead of a purified component system. Transcription was initiated with limiting NTPs including α^32^P-CTP. The initial incubation mix and unbound nuclear extract proteins were washed out with transcription buffer, and the engaged polymerases were then chased with nonradioactive NTPs. Transcripts were resolved on denaturing gels.

### Nucleosomes downstream of the TSS inhibit transcription

**Figure 2:**
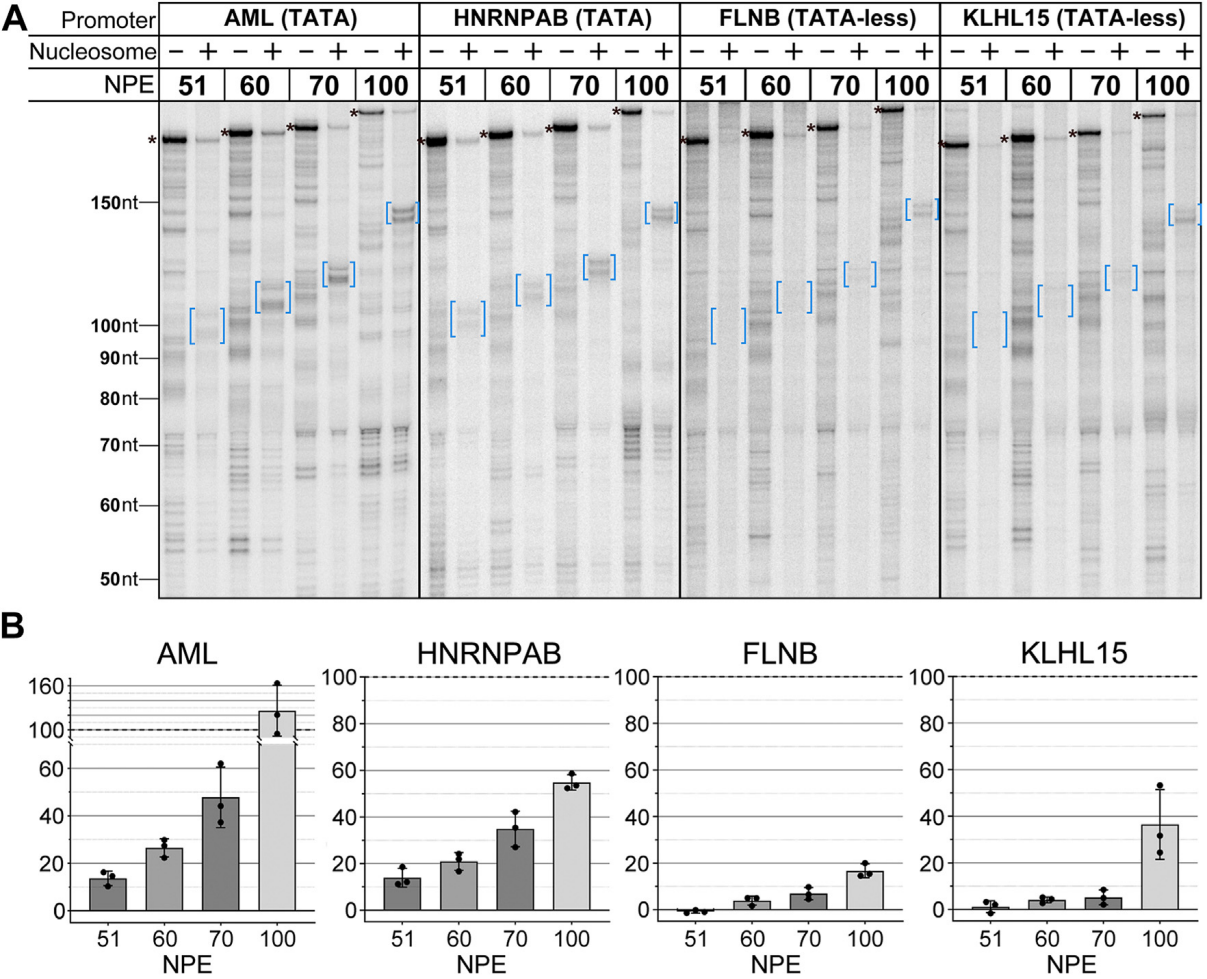
**A nucleosome with a proximal edge at +51 is strongly (TATA promoters) or almost completely (TATA-less promoters) inhibitory to transcription.***A*, four templates with the indicated promoters were transcribed as free DNA or with NPEs at the indicated locations. Transcripts on the nucleosome templates mostly extend to only 45 to 55 bp within the nucleosome (indicated with *blue brackets*). Numbers on the *left* indicate the lengths of the RNAs, which were validated with size markers run in adjacent lanes. The positions of run-off RNAs are indicated by the *asterisks*. Lengths of these RNAs are 238, 247, 257, and 287 for the 51, 60, 70, and 100 templates, respectively. *B*, graphs show signals at the nucleosome barriers compared with the runoff signal from the non-nucleosomal samples (barrier/runoff × 100%); n = 3, error bars are ±1 standard deviation. NPE, nucleosome proximal edge.

As shown in Figure 2*A*, all the non-nucleosomal templates were strongly transcribed, including the two TATA-less promoters. We were surprised to find that the nucleosomal 51 templates were either substantially less active (AML and HNRNPAB) or inactive (FLNB and KLHL15) for transcription. This is in sharp contrast to our earlier studies with nucleosomal TATA-promoter templates in which the TSS to nucleosome distance was 50 bp (27, 28, 29). However, in those experiments, transcription complexes were assembled using purified transcript initiation factors including TBP only, not TFIID. The loss of transcripts in the 51 template lanes was not apparently accompanied by an accumulation of shorter RNAs. This suggests that the inactivity of these templates reflects a failure to assemble PICs, though we would not have detected RNAs shorter than ∼10 nt. As the NPE is moved downstream, transcription increased for the TATA promoters, with at least half of the naked DNA signal restored for the 100 templates. In contrast, substantial transcription was only observed for the TATA-less 100 templates (Fig. 2*B*). On all templates, the large majority of polymerases that did initiate advanced to two closely spaced major pause points ∼45 to 55 bp within the nucleosome, marked by brackets on each panel. This is consistent with many earlier studies on the barrier imposed to advancing pol II by nucleosomes located on the 601 element and on other positioning sequences (27, 30, 31). Importantly, the major nucleosomal pause locations are identical for all four promoters, consistent with our expectation that the three native promoters, both TATA and TATA-less, support initiation from a single TSS.

### TBP supplementation reverses transcription inhibition on TATA templates only

**Figure 3:**
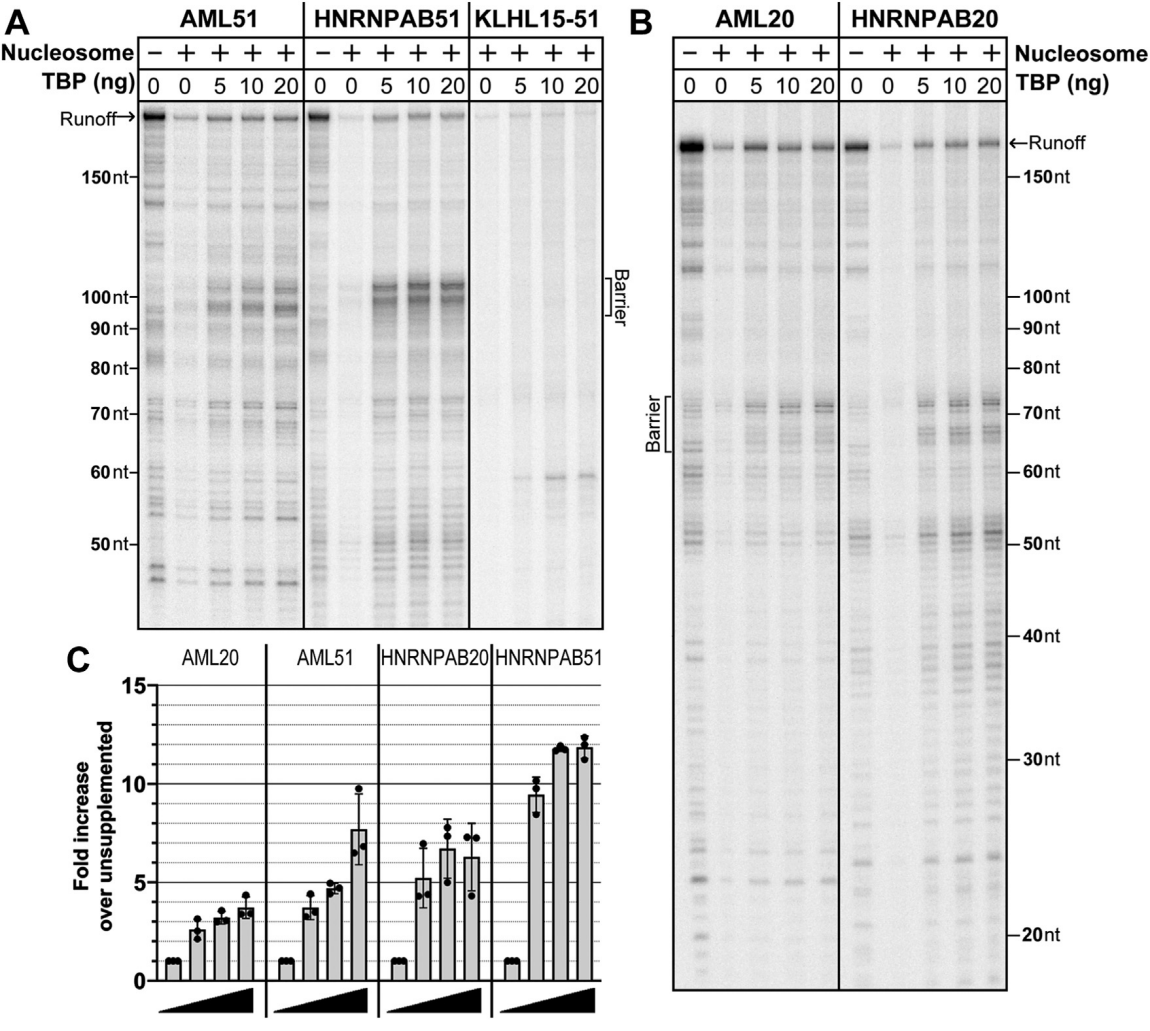
**Transcriptional activity is restored on poorly active or inactive nucleosomal templates by supplementation with TBP on TATA promoters.***A*, addition of increasing levels of TBP restores transcription on the NPE +51 TATA promoter templates but not on the +51 TATA-less KLHL15 template. *B*, increasing levels of TBP partially restores transcription on NPE +20 TATA promoter templates. *C*, graph shows fold enhancement over unsupplemented reactions (TBP supplemented/unsupplemented) of the transcript at the barrier 45 to 55 bp within the nucleosome. n = 3, error bars are ±1 standard deviation. NPE, nucleosome proximal edge; TBP, TATA-binding protein.

In considering the basis for inhibition of transcription on the nucleosomal templates, we focused on the differences in response of the TATA and TATA-less templates to the proximal nucleosome. Assembly of the PIC at TATA-less promoters depends on elements downstream of the TSS, most recently mapped at +17 to +35 (8), whereas assembly at TATA promoters also relies on the TATA element and may be driven by TATA alone (4, 16). A previous report on *in vitro* transcription of nucleosomal templates (24) using TFIID to support PIC assembly showed that an NPE at +39 substantially reduced production of RNA compared with pure DNA templates. Since an earlier study demonstrated that the TFIID requirement can be bypassed on TATA promoters with TBP alone (32), supplementing our nuclear extract reactions with TBP could support efficient transcription on nucleosomal TATA element promoters by circumventing potential limitations of TFIID–template interactions imposed by the downstream nucleosome. An initial test with the 51 templates showed that this is the case (Fig. 3, *A* and *C*): TBP supplementation for either of the TATA promoters does restore transcriptional activity, nearly to that level seen with the naked DNA template. Addition of the same amounts of TBP did not rescue activity on the TATA-less KLHL15 51 promoter (Fig. 3*A*).

The TBP-dependent activity of the 51 TATA templates raises the question of how close the +1 nucleosome can approach the TSS while retaining transcriptional activity. Even when PIC assembly is driven primarily by interactions with the TATA element upstream, recent evidence indicates that downstream contacts are still involved at TATA promoters, primarily from the XPB subunit of TFIIH (3, 4). Accordingly, we constructed a series of templates based on the HNRNPAB promoter with TSS to NPE spacing of 45, 40, 35, 30, and 20 base pairs. The reported structures of TBP-based PICs suggested that PIC assembly on the 45, 40, and 35 templates would not be blocked by the nucleosome, but the 30 and particularly 20 templates would not support transcription (3). However, initial TBP titrations as in Figure 3*A* showed that all these templates were transcriptionally active. To confirm that the surprising 20 template result was not specific to HNRNPAB, we also constructed an AML 20 template. Both these 20 templates reproducibly supported about half the activity of the 51 templates with the same promoter (Fig. 3, *B* and *C*). The significance of this observation for mechanistic understanding of XPB-driven template melting will be explored in the Discussion section.

The results in Figures 2 and 3 strongly suggest that differences in loading TFIID for TATA and TATA-less promoters account for the much greater inhibitory effect of a proximal nucleosome in the absence of a TATA element. To demonstrate the role of TFIID directly at both promoter classes in our extract-based transcription system, we took advantage of earlier observations that heat treatment of nuclear extracts selectively inactivates TFIID (33, 34). Heat treatment of our extract causes complete loss of transcriptional activity on both the HNRNPAB TATA promoter and the KLHL15 TATA-less promoter (Fig. S1). Activity is robustly restored on the TATA promoter in the treated extract by addition of TBP, but TBP addition does not rescue the TATA-less promoter. Transcription of both the TATA and TATA-less promoters must therefore depend on TFIID in (nontreated) extract-based reactions, as we had supposed. Rescue of transcription by TBP on the TATA promoter in nontreated extracts (Fig. 3*A*) should result from bypassing TFIID. It seems unlikely that the transcriptional stimulation from added TBP is caused by the assembly of additional TFIID by combination of the added TBP with free TAFs in the extract, since TBP addition allows transcription of the otherwise inactive TATA 20 templates (Fig. 3*B*). On those DNAs, the NPE would completely occlude the downstream promoter elements required for TFIID binding, which extend out to about +35. A +1 nucleosome with a proximal edge at 51 is strongly (TATA promoters) or completely (TATA-less promoters) inhibitory to transcription when those promoters depend on TFIID, but a +51 TATA template is transcribed at near-naked DNA levels when driven only by TBP (Fig. S1).

### Nucleosomes with H2A.Z allow more traversal than nucleosomes with H2A

**Figure 4:**
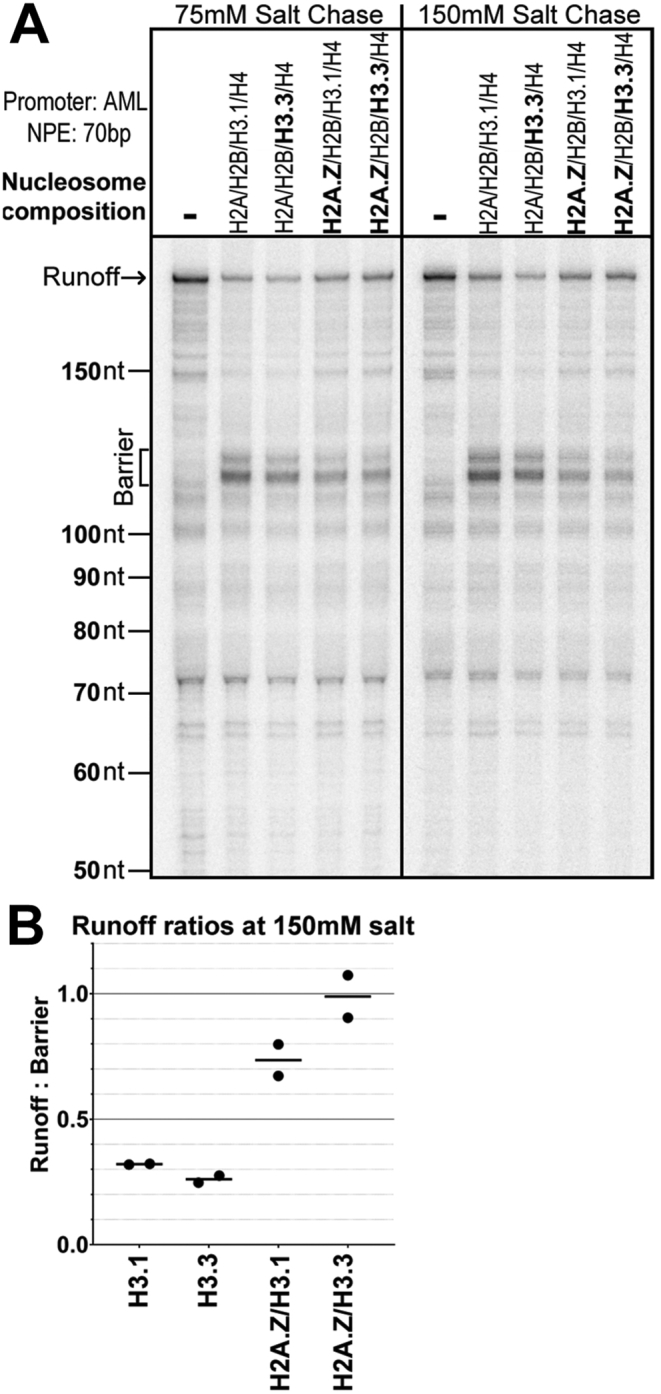
**Nucleosome traversal is slightly facilitated with nucleosomes containing H2A.Z.***A*, AML promoter NPE +70 templates were transcribed with nucleosomes having the indicated compositions and total salt at 75 or 150 mM in the chase reactions. *B*, graph compares the ratio of the runoff to the 45 to 55 barrier signal as a measure of traversal efficiency for the 150 mM salt chases. Ratios per experiment are plotted as *dots*, *horizontal bars* show the mean, n = 2. AML, adenovirus major late; NPE, nucleosome proximal edge.

Nucleosomes at active promoters are often enriched for particular histone variants and post-translational modifications. We reconstituted nucleosomes with combinations of two of these variants, H2A.Z and H3.3, and compared their effects on transcription on AML 70 templates. We did not detect an effect on overall transcription levels among any of the combinations of histone variants tested (Fig. 4*A*). However, there was a modest effect on the level of nucleosome traversal (Fig. 4*A*). This was only observed for nucleosomes containing H2A.Z and was more apparent when the reactions were chased in 150 mM salt instead of 75 mM used in all other experiments (Fig. 4*A*, compare *left* and *right panels*). The traversal increase was approximated by comparing the signal at the major nucleosome barrier to the signal of the run-off products. In reactions chased at 150 mM with nucleosomes containing the usual histone complement, the level of run-off RNA to major pause RNA was about 1:3, but for nucleosomes containing H2A.Z, that ratio increased to nearly 1:1 (Fig. 4*B*). This increase in traversal is consistent with the recent observation that incorporation of H2A.Z promotes nucleosome unwrapping (35).

### Promoter-proximal nucleosomes with H3K4me3 restore transcription

**Figure 5:**
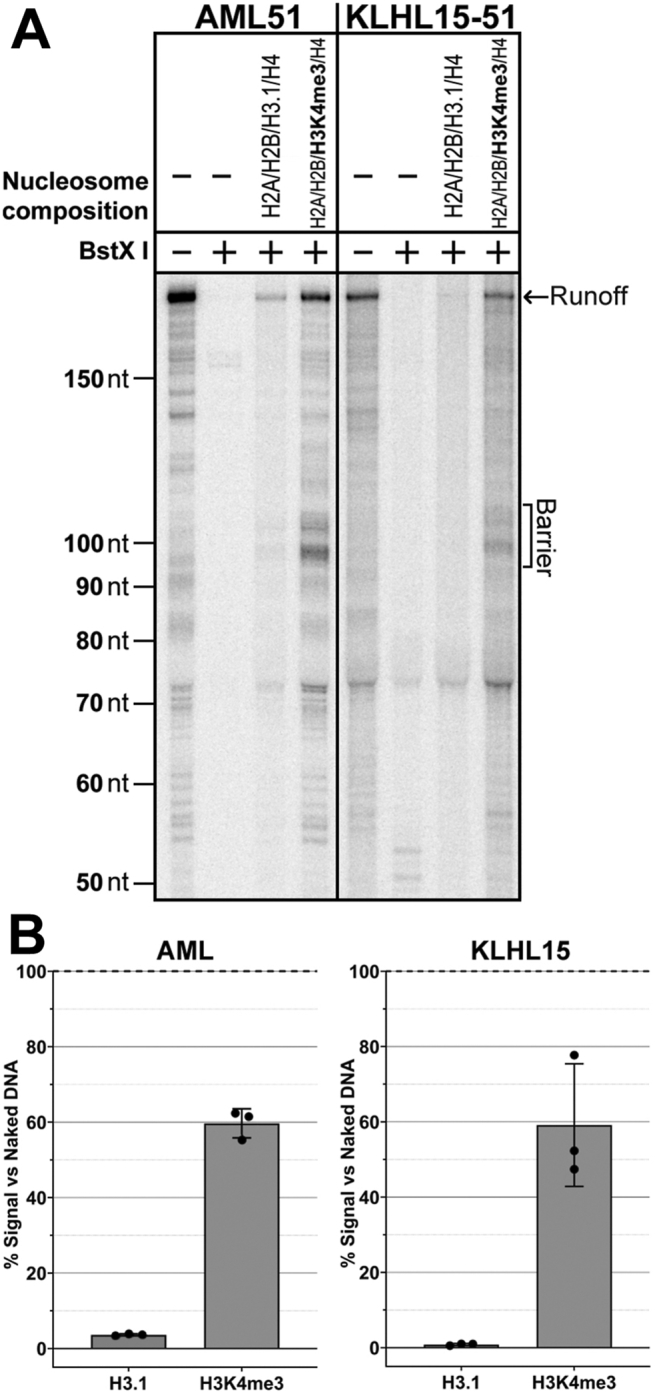
**Activity on nucleosomal templates can be restored by substituting H3 with H3K4me3 for TATA and TATA-less promoters.***A*, NPE +51 templates with the indicated promoters and nucleosome compositions were transcribed without TBP supplementation after cleavage of the templates with BstXI, which releases templates lacking nucleosomes. Transcriptions of DNA templates without BstXI cleavage are shown in the *leftmost lane* of each set for comparison. *B*, graphs show signals at the nucleosome 45 to 55 barriers compared with the runoff signal from the non-nucleosomal samples (barrier/runoff × 100%); n = 3, error bars are ±1 standard deviation. H3K4me3, histone H3 trimethylated on lysine 4; NPE, nucleosome proximal edge; TBP, TATA-binding protein.

Certain histone modifications are known to be enriched at active promoters. Trimethylation of lysine 4 of H3 is of particular interest, since it has been shown that TAF3 of TFIID interacts specifically with this modified histone (20). We assembled nucleosomes on 51 templates containing H3K4me3 with either AML (TATA) or KLHL15 (TATA-less) promoters. The nucleosome positioning sequences for these templates were slightly modified to incorporate a unique BstXI restriction site. We treated the bead-attached templates with BstXI followed by rinsing to ensure that any residual non-nucleosomal DNA was eliminated. The presence of nucleosomes lacking the H3K4me3 modification greatly reduced transcription on these templates. However, when the H3K4me3 mark was present, transcription was restored for both TATA (AML) and TATA-less (KLHL15) promoters (Fig. 5).

## Discussion

### Promoter-proximal nucleosomes inhibit transcription by interfering with TFIID

We have studied the effect of nucleosomes located immediately downstream of the promoter on pol II transcription *in vitro* using nuclear extracts. Our experiments included native promoters of both the TATA and TATA-less classes, a range of +1 nucleosome locations similar to those seen *in vivo*, and a number of histone types and modifications correlated with promoters. Our primary conclusion is that the +1 nucleosome acts to inhibit transcription by interfering with the loading of TFIID at the promoter. Selective inactivation of TFIID in extracts demonstrates that both promoter classes rely on TFIID to support transcription in extracts (Fig. S1). Only TATA promoters recover activity in the TFIID-depleted extracts when TBP is added. A proximal nucleosome has a greater negative effect on transcription for TATA-less promoters, where PIC assembly depends on TFIID contacts downstream of the TSS, than on TATA promoters where PIC assembly can also be supported by TBP interaction with the upstream TATA element (Fig. 2). Driving promoter function at TATA promoters by addition of TBP relieves nucleosomal inhibition (Figs. 3 and S1). As expected, TATA-less promoters are not responsive to TBP supplementation. A schematic summary of our results is shown in Fig. S3. Crucially, inhibition was relieved on both promoter classes when H3K4me3 was substituted for H3 in the +1 nucleosome (Fig. 5). The effect of the modification is consistent with the expectation that facilitating TFIID loading though the reported TAF3–H3K4me3 interaction (20, 36) should overcome the nucleosomal inhibition.

In our earlier work with nucleosomal templates, we showed that an NPE at +50 did not inhibit transcription by pol II from an AML promoter when transcription was supported with TBP and other purified general factors (27, 28). In light of those results, we were initially surprised by the strong inhibition of transcription imposed by the nucleosomes in our extract-based reactions. We stress that this transcriptional blockade does not simply reflect a general repression of transcription in extracts, since 51 templates with TATA promoters are essentially as active as pure DNA templates with TBP supplementation. One plausible explanation involves the location of the template sequences most important for TFIID loading on TATA-less promoters, which extend from roughly +17 to +35 (4, 8). If TFIID reaches these critical contacts by approaching from downstream, the presence of a +1 nucleosome without any additional support for TFIID loading could be strongly inhibitory. This also predicts that as we observed, nucleosomal TATA promoters without supplemental TBP will be at least partly repressed relative to pure DNA in extracts since in this case TBP can access the TATA element only as a part of TFIID. Relevant in this context, a recent report showed that the average TSS to +1 nucleosome distance does not differ between TATA and TATA-less promoters (9).

### Transcription can be conditionally restored on templates with nucleosomes close to the TSS

In a major earlier study, Nock *et al.* (24) assembled mononucleosome templates with four copies of an HNF4-cognate binding site upstream of an AML promoter and varying distances from the TSS to the NPE. In those assays, transcription was driven with purified pol II and general factors including TFIID as well as HNF4α, PC4, and in some cases mediator or chromatin cofactors. Comparisons between their activator-assisted factor-based system and our studies in extracts are problematic, but it is worth noting in particular that templates in the earlier study with the NPE at +39 supported substantially lower levels of transcription relative to the pure DNA controls, similar to the ∼5-fold reduction we observed on 51 TATA chromatin templates compared with the DNA-only controls (Fig. 2). Another previous study explored the effects of substituting H3K4me3 for H3 in nuclear extract–based reactions on multinucleosomal templates, using the AML promoter to drive transcription (36). The experimental design in those studies was much more complex than ours, and the +1 nucleosomes did not have a fixed location relative to the TSS. Nevertheless, it is relevant that in that case, H3K4me3 nucleosomal templates supported ∼5-fold more transcription than H3 nucleosomal templates. The increase in transcription with H3K4me3 was maintained in that earlier study when the TATA element was mutated from TATAA to TATGG (36). Native TATA-less promoters were not assayed in either of the experiments just cited. A very recent study reported that the presence of a +1 nucleosome can stimulate transcription through interactions between mediator and the nucleosome. This stimulation depended on specific mediator residues and was observed on templates with an NPE at +40 but not at +70 (37). All these results support the central idea that the +1 nucleosome can actively facilitate transcription complex assembly.

### An NPE 20 bp downstream of the TSS still allows assembly of an active TFIID-independent PIC

Our finding that the 20 TATA templates are active with TBP addition adds to our understanding of the transition from PIC to open complex and ultimately to promoter clearance driven by XPB. After TFIIH joins the nascent PIC, the interaction of XPB with DNA extends downstream to approximately +20, displacing the TAFs from that section of the template (4). In the presence of ATP, XPB translocates DNA upstream into the PIC to cause template melting (13). Formation of the initial transcription bubble requires unwinding of ∼9 bp (38); after initiation, the bubble increases to ∼17 to 18 bases at which point, the activity of XBP is no longer required (38, 39). Thus, XPB translocates as much as 17 bp of DNA from downstream of its original position on the template to drive the complete transition from PIC to open complex and subsequent promoter clearance. Earlier work showed that transcript initiation at the AML promoter using TBP and purified factors requires at least 36 bp of template DNA downstream of the TSS (40). Since nearly all that necessary DNA must be acquired from the adjacent nucleosome on our active 20 TATA templates, the translocase activity of XPB is sufficiently powerful to remove DNA initially bound to the proximal H2A–H2B dimer of the +1 nucleosome. This is apparently more difficult than translocating non-nucleosomal DNA because the 20 TATA templates are less active than the 50 TATA templates (Fig. 3). Nock *et al.* (24) tested a template in their factor-based system with an NPE at +19. That template was inactive with TFIID, but some activity was restored when TBP was substituted. This reinforces the importance of contacts downstream of +20 for the function of TFIID, even in the presence of mediator and multiple upstream-binding sites for activators. The effectiveness of TBP-based transcription on the +19 template was not compared with the +39 template with TBP or with pure DNA in those earlier assays.

### Promoter-proximal nucleosomes actively regulate transcription through TFIID

A nonmodified +1 nucleosome substantially or completely inhibits *in vitro* transcription by pol II in nuclear extracts depending on promoter class, location of the nucleosome, and bypass of TFIID with added TBP (Fig. S3). *In vivo* measurements of nucleosome positions suggest that 51 templates should be fully permissive for transcription complex assembly (9), given the known dimensions of the PIC. Our results argue that the +1 nucleosome is not a passive bystander; instead, that nucleosome can be regarded as a regulator of transcription complex assembly. Proximal nucleosomes lacking activating modifications disrupt productive association of TFIID with the promoter, whereas nucleosomes containing activating modifications stabilize TFIID association and facilitate PIC assembly. If optimizing PIC–nucleosome interaction is needed for efficient PIC assembly, it should also be important that the +1 nucleosome be located as close as possible to the nascent PIC without actually encroaching on the footprint of TFIID. This is consistent with the recent observation that DNA sequences extending from 50 to 200 bp downstream of human TSSs should favor positioning of the +1 nucleosome to that same location (9). In this context, it is tempting to speculate that the bias in metazoan promoters for a TATA-less architecture (1), which requires TFIID interaction with DNA elements downstream of the TSS, also favors the presence of an immediately downstream nucleosome to provide positive interactions with the transcriptional machinery.

## Experimental procedures

### DNA sequences

DNA sequences used for nucleosome reconstitution were based on the clone 601 nucleosome positioning element (26) with some modifications. Two segments of the sequence containing poly-AT tracks were substituted with the corresponding sequences of clone 603, and a BstXI site was introduced on the promoter-distal portion of the sequence by single nucleotide substitution (C→T). The complete sequence, 601Sb (shown in Table S1),was ordered as a gBlock (IDT), cut with HindIII and EcoRI, and cloned into the polylinker of pUC19 using the same restriction sites. DNA primers 601R_AvaI and 601R_Rev_Biot (IDT; shown in Table S1) were used to amplify the nucleosome positioning sequence from the pUC19 plasmid for the DNA template fragment preparation.

The AML DNA template sequence is centered on the core element from −41 to +10 of the AML promoter as adapted from previous work (27). The complete sequence (shown in Table S1) was cut with BamHI and HindIII and cloned into the polylinker of pUC19 cut with the same restriction enzymes. The sequence used for the HNRNPAB promoter corresponds to human genome chr5: 178204390 to 178204665 (GRCh38/hg38) with the downstream end modified into a SacI site by point mutation (T->C). It was ordered as a gBlock (IDT; shown in Table S1), cut with XbaI and SacI, and cloned into the polylinker of pUC19 cut with the same restriction enzymes. Sequence for the FLNB promoter corresponding to human genome chr3: 58008029 to 58008588 (GRCh38/hg38) was PCR amplified from human genomic DNA from leukocytes (Roche) using DNA primers FLNB_U and FLNB_D (IDT; shown in Table S1), cut with SacI and XbaI, and then cloned into the corresponding sites in the polylinker of pUC19. Sequence for the KLHL15 promoter corresponding to human genome chrX: 24025050 to 24025555 (GRCh38/hg38) was PCR amplified from human genomic DNA from leukocytes (Roche) using DNA primers KLHL15_U and KLHL15_D (IDT; shown in Table S1), cut with SacI and XbaI, and then cloned into the corresponding sites in the polylinker of pUC19. An AvaI site in the cloned sequence was eliminated by mutating a single base at −23 from the TSS (C->G).

Promoter DNA fragments were amplified from the cloned sequences. The distances from the TSS to the NPE of promoter DNA fragments were determined by the downstream primers used. The upstream primer for AML was M13R, and the downstream primers were AML20_AvaI, AML51_AvaI, AML60_AvaI, AML70_AvaI, and AML100_AvaI (IDT; sequences are shown in Table S1). The upstream primer for HNRNPAB was HNRNPAB-96, and the downstream primers were HNRNP20_AvaI, HNRNP51_AvaI, HNRNP60_AvaI, HNRNP70_AvaI, and HNRNP100_AvaI (IDT; sequences are shown in Table S1). The upstream primer for FLNB was FLNB_Upstream, and the downstream primers were FLNB51_AvaI, FLNB60_AvaI, FLNB70_AvaI, and FLNB100_AvaI (IDT; sequences are shown in Table S1). The upstream primer for KLHL15 was M13F, and the downstream primers were KLHL15-51_AvaI, KLHL15-60_AvaI, KLHL15-70_AvaI, and KLHL15-100_AvaI (IDT; sequences are shown in Table S1).

### DNA template fragment preparation

DNA fragments were PCR amplified from plasmids in 800 μl volumes using a Q5 High-Fidelity PCR kit (NEB) following the manufacturer’s recommended procedure. After amplification, the reaction was then passed through a single column from a Monarch PCR & DNA cleanup kit (NEB) following the manufacturer’s recommendations for double-stranded DNA <2 kb. The column was eluted with 15 μl elution buffer and digested with 10 U AvaI at 37 °C for 2 h. For KLHL15 promoter fragments only, before digesting with AvaI, the eluate was digested with 10 U SmaI at 37 °C for 1 h followed by treatment with 5 U QuickCIP (NEB) for 30 min at 37 °C and heat inactivation for 10 min at 80 °C. The reactions were stopped by adding 4 μl 6× Purple Gel Loading Dye (NEB) and then run on a 0.8% Tris:acetate:EDTA agarose gel. Fragments were gel purified using a QIAquick gel extraction kit (Qiagen) according to the manufacturer’s recommendations and were further concentrated by ethanol precipitation.

### Nucleosome reconstitution

Histone tetramers containing H4 and H3K4me3 were custom ordered from EpiCypher. All other histone dimers, tetramers, and octamers were purchased from ActiveMotif. Nucleosomes were reconstituted using a microscale reconstitution method performed at 24 °C adapted from previous work (25) with the following modifications: each 10 μl reaction contained 500 ng DNA, 2 M NaCl, 10 mM Tris (pH 7.5), 5 mM β-mercaptoethanol, 0.2 mM EDTA, and 0.05% Igepal CA-630. The dilution buffer contained 10 mM Tris (pH 7.5), 5 mM β-mercaptoethanol, 0.2 mM EDTA, and 0.05% Igepal CA-630. The exact ratio of histones to DNA varied between histone lots and required titration but was between 1.1:1 and 1.7:1 histone octamer to DNA for any lot. Histone dimers and tetramers were added at 2:1 dimer:tetramer ratios where required. Salt reductions were performed by adding dilution buffer followed by a 1 h incubation at 24 °C after each addition. The volumes of dilution buffer added were 10 μl (reaction diluted to 1 M NaCl), 5 μl (800 mM NaCl), 5 μl (667 mM NaCl), 70 μl (200 mM NaCl), and 100 μl (diluted to 100 mM NaCl). Reconstitutions were assessed by 4.5% native PAGE and ethidium bromide staining. Representative examples of the native gels are shown in Fig. S2. Only reconstitutions with minimal amounts (<1%) of naked DNA were used for template assembly.

### Template assembly

Templates were ligated by mixing promoter DNA fragments with 100 ng reconstituted nucleosome fragments at 2:1 M ratios followed by addition of 10 μl 10× T4 DNA ligase buffer (NEB) and 400 U T4 DNA ligase (NEB) in a final volume of 100 μl. The reactions were incubated for 16 h at 15 °C and diluted to 500 μl with TE100 buffer (10 mM Tris [pH 7.5], 100 mM NaCl, and 1 mM EDTA). About 15 μl of Dynabeads M-280 streptavidin (Invitrogen) were washed according to the manufacturer’s recommendations and resuspended in 15 μl of TE100. These resuspended beads were added to the diluted ligation reaction, gently mixed, and incubated at 24 °C for 2 min. The beads were then washed twice with 100 μl TE250 (10 mM Tris [pH 7.5], 250 mM NaCl, and 1 mM EDTA) and resuspended in 35 μl TE100. Successful ligation of templates was verified by phenol:chloroform extracting 5 μl of beads and resolving the ethanol-precipitated DNA on a 4.5% native PAGE. The gel was stained with ethidium bromide and imaged using a GelDoc EZ (Bio-Rad). Bands were quantified using Image Lab, version 6.1.0. Where BstXI digestion was necessary, the beads were resuspended in 50 μl 1× r3.1 buffer (NEB) and incubated at 37 °C for 1 h with 10 U BstXI. After digestion, the beads were washed twice with 100 μl TE250 and then resuspended in 35 μl TE100.

### Transcription assay

Preinitiation complexes were assembled for 45 min at 30 °C with the following per 25 μl reaction: 50 ng of bead-conjugated template, 0.5 μl RNasin Plus (Promega), 9 μl of HeLa cell nuclear extract (prepared as described (41)), and 250 ng HaeIII cut *Escherichia coli* DNA. Final buffer for PIC assembly was 20 mM Tris (pH 7.9), 1 mM DTT, 8 mM MgCl_2_, 35 mM KCl, 40 mM NaCl, 7% glycerol, and 0.5 mM EDTA. Reactions were initiated by adding 3.5 μl of limiting NTPs to a final concentration of 100 μM ATP, 0.767 μM α^32^P-CTP (PerkinElmer), and 60 μM UTP (for AML templates only) or 60 μM GTP (for HNRNPAB, FLNB, and KLHL15 templates), followed by incubation at 30 °C for 2 min. The reactions were then washed twice with 70 μl XM75 buffer (20 mM Tris [pH 7.9], 1 mM DTT, 8 mM MgCl_2_, 35 mM KCl, 40 mM NaCl, 7% glycerol, 0.5 mM EDTA, 100 ng/μl bovine serum albumin, 5 ng/μl sonicated herring sperm DNA) supplemented with 0.002% Igepal CA-630, changing to a fresh test tube on the second wash. Reactions were resuspended in 28.5 μl XM75 and then chased by adding 3 μl of 2 mM each of ATP, CTP, GTP, and UTP to bring each NTP concentration to 190 μM, followed by incubating at 30 °C for 5 min. Reactions that were chased at 150 mM salt were resuspended in XM150 instead of XM75 (70 mM KCl, 80 mM NaCl instead of 35 mM and 40 mM, respectively). Reactions were stopped by adding 70 μl of stop solution (20 mM EDTA, 200 mM NaCl, 1% SDS, 100 μg/ml proteinase K, and 200 μg/ml tRNA) and incubated at 30 °C for 5 min. For the experiments that were supplemented with TBP (prepared as described (39)), reaction volumes were half of what is specified previously. Reactions were supplemented with an additional 1 μl of BC100 or the amounts of TBP in BC100 buffer as specified in Figure 3 or Fig. S1 before incubating to form a PIC. For heat-inactivation assays, HeLa nuclear extract was incubated at 45 °C for 15 min (34) before use in PIC assembly as aforementioned. Stopped reactions were phenol:chloroform:isoamyl alcohol extracted, ethanol precipitated, and resuspended in 7 M urea sequencing gel loading dye. Transcripts were heated to 95 °C for 3 min, incubated on ice for 2 min, and then run on a 7 M urea 10% polyacrylamide sequencing gel for 90 min at 45 W constant power. Dried gels were exposed to phosphorimager screens, which were scanned using a GE Amersham Typhoon scanner. Images were analyzed with ImageQuant TL software.

## Data availability

All data that support this study are provided in the article.

## Supporting information

This article contains supporting information.

## Conflict of interest

The authors declare that they have no conflicts of interest with the contents of this article.
