# Supplementary material for: Promoter-proximal nucleosomes attenuate RNA polymerase II transcription through TFIID
Source: J Biol Chem. 2023 Jun 15;299(7):104928. doi: 10.1016/j.jbc.2023.104928 (PMC10404688; doi:10.1016/j.jbc.2023.104928)
Supplement: Supplemental Figure S1 [file mmc1.pdf]

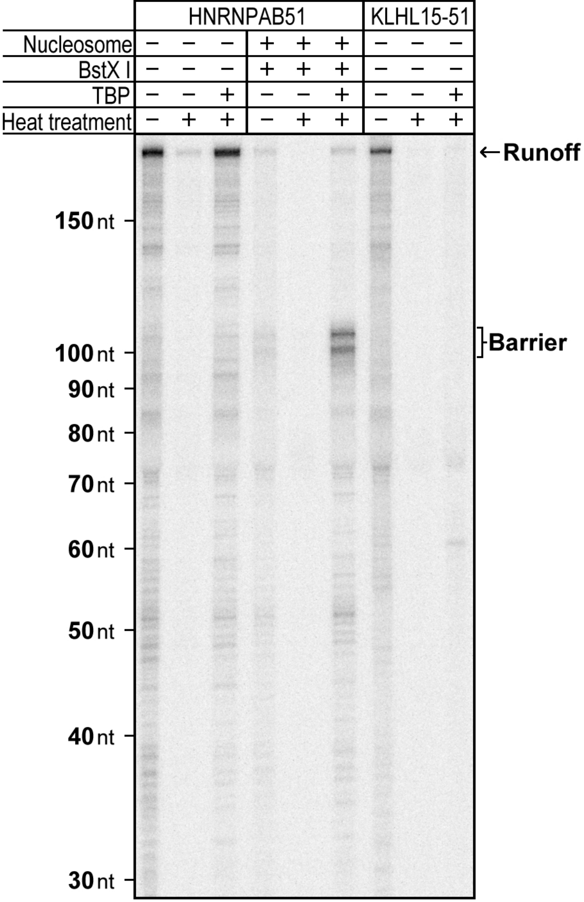

Fig. S1 TBP restores transcription activity for heat-treated nuclear extracts but only on TATA promoters. TFIID can be selectively inactivated by heat-treatment at 45°C for 15 minutes. Where indicated, reactions were supplemented with 10ng TBP. Transcription was restored for TBP supplemented reactions using the HNRNPAB promoter (TATA) on naked DNA or with a nucleosome with an NPE at +51, but not using the KLHL15 promoter (TATA-less).
