# Supplementary material for: Promoter-proximal nucleosomes attenuate RNA polymerase II transcription through TFIID
Source: J Biol Chem. 2023 Jun 15;299(7):104928. doi: 10.1016/j.jbc.2023.104928 (PMC10404688; doi:10.1016/j.jbc.2023.104928)
Supplement: Supplemental Figure S2 [file mmc2.pdf]

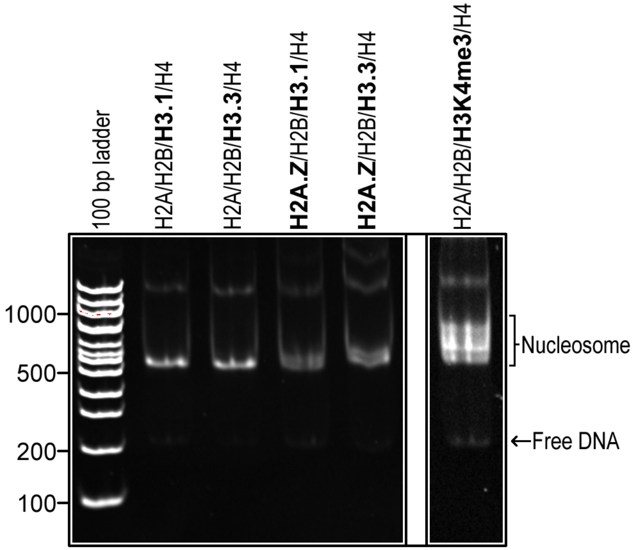

Fig. S2 Native PAGE gel of reconstituted nucleosomes. Samples of each reconstitution are run on a 4.5% 29:1 acrylamide:bis native gel and stained with ethidium bromide. Naked DNA runs slightly above 200bp (indicated by an arrow) and is only slightly visible at this exposure. Reconstituted nucleosomes shift the DNA into the 600-900bp range (indicated by a bracket). Molecular weight markers are a 100bp DNA ladder (NEB).
