# Supplementary material for: Promoter-proximal nucleosomes attenuate RNA polymerase II transcription through TFIID
Source: J Biol Chem. 2023 Jun 15;299(7):104928. doi: 10.1016/j.jbc.2023.104928 (PMC10404688; doi:10.1016/j.jbc.2023.104928)
Supplement: Supplemental Figure S3 [file mmc3.pdf]

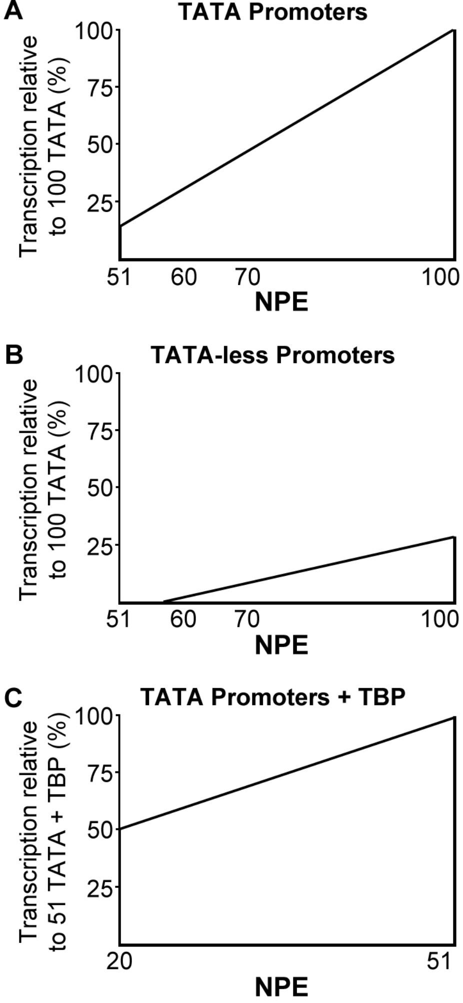

Fig. S3 Schematic representation of effects of a downstream nucleosome on transcription initiation for TATA-less and TATA-containing promoters. (a) On TATA promoters, transcription activity decreases as a nucleosome is moved closer to the TSS. (b) Transcription activity on TATA-less promoters is more strongly inhibited by a downstream nucleosome than for TATA promoters, becoming undetectable with an NPE at +51. (c) Supplementation with TBP restores transcription activity on nucleosomal templates but only for TATA promoters. Transcription levels decrease as the nucleosome is moved closer to the TSS, but can still occur with an NPE as close as 20bp.
