# Supplementary material for: Promoter-proximal nucleosomes attenuate RNA polymerase II transcription through TFIID
Source: J Biol Chem. 2023 Jun 15;299(7):104928. doi: 10.1016/j.jbc.2023.104928 (PMC10404688; doi:10.1016/j.jbc.2023.104928)
Supplement: Supplemental Table S1 [file mmc4.pdf]

601Sb	TACTCTCGGGCGCCAAGCTTACAGGATG**<u>GACTTGA</u>**CTGACACGTGCCTGGAGACTAGGGAGTA ATCCCCTTGGCGGTT**<u>GTTAGCCTTGGATTG</u>**GCGCGTACGTGCGTTTAAGCGGTGCTAGAGCTGTCTA CGACCAATTGAG**<u>T</u>**GGCCTCGGCACCGGGATTCTCCAGCCCCGTTTCCGGATCCCCGGATACCGAGC TCGAATTCACTAG
AML	AAGCTTAGCTTCCGGAAGGGGGGCTATAAAAGGGGGTGGGGGCGCGCTCGTCCTCACTCTCTTCCCC TTCTCTTTAAAGGCCTTTGGGAAACCCTCGGCACTGGGTACCCAGGGACTTGAAGTAATAAGGACGG AGGGCCTCTTTCAACATCGATGCACGGTGGTTAGCCTTGGATTGCCCTCTACCGTGGCCTAAGCGTAC TTAGAAGCCCGAGTGACGACTTCACACGGTAGGTGGGCGCGCGAACTGGGCCCCGTTTCCGGATCC
HNRNPAB	CCAGGTCTAGAGCGCGCGCGCCGTGGCGGCCAATGGGACGCTGAAGCTAGGTTGAGTGACGGACTT GAGCAGCCAATGAGTGTGCAGTTTGTTGCGATGTGGCACCCGGCTCCGGCATTATAAAGGGCGCCAC GAGTCGGCATTGTCAGGCGGCGGCACCGCGCGGGACGGAGCTTGGCTGTTGGTCGGTGGGTTCCCGT GCGGCGGCGGCCAAGGAGGAGGAGACACAGTTGGAGCAGCTCCGTGGGCTGACTGGGGCGAGGCC TCAGCAGCGCGAGCTCGAGTGC
601R_AvaI	TACTCTCGGGCGCCAAGCTTACAGGATG
601R_Rev_Biot	5'-Biotin - AGTGAATTCGAGCTCGGTATCCGGGGATCCG
FLNB_U	GAATGGCTGGTACACAGGCGGAGCTC
FLNB_D	CCTCATCTAGATCCTTCTCGGTTACCGGCATC
KLHL15_U	CGGGCGAGCTCCCCGCCCCCAACGGCTTCCCAC
KLHL15_D	CCCCTCTAGATAAGTCCTGGGAAAGAAGACAGC
M13R	CAGGAAACAGCTATGACCATG
AML20_AvaI	TACTCCCGAGGAGTGAGGACGAGCG
AML51_AvaI	TACTCCCGAGTTCCCAAAGGCCTTTAAAGAG
AML60_AvaI	TACTCCCGAGGCCGAGGGTTTCCC
AML70_AvaI	TACTCCCGAGGGTACCCAGTGCCGAG
AML100_AvaI	TACTCCCGAGCCCTCCGTCCTTATTACTTC
HNRNPAB-96	TACTAAGCTTGAGTGACGGAC
HNRNP20_AvaI	TACTCCCGAGGCCTGACAATGCCGAC
HNRNP51_AvaI	TACTCCCGAGACAGCCAAGCTCCGTC
HNRNP60_AvaI	TACTCCCGAGCACCGACCAACAGCC
HNRNP70_AvaI	TACTCCCGAGCACGGGAACCCACCG
HNRNP100_AvaI	TACTCCCGAGACTGTGTCTCCTCCTCCTTG
FLNB_Upstream	GCCCTACATCCTGCTGGG
FLNB51_AvaI	TACTCCCGAGCTTGCTGCTACCGGAG
FLNB60_AvaI	TACTCCCGAGGGGTTCGAACTTGCTG
FLNB70_AvaI	TACTCCCGAGAGCGGGAGCGGGGTTC
FLNB100_AvaI	TACTCCCGAGCAAGGGCCGAAGGAGCG
M13F	TTGTAAAACGACGGCC
KLHL15-51_AvaI	TACTCCCGAGCGAGGAAGCGTCGAG
KLHL15-60_AvaI	TACTCCCGAGAGCACCAGCCGAGGAAG
KLHL15-70_AvaI	TACTCCCGAGCAGAGACAGAAGCACCAGCC
KLHL15-100_AvaI	TACTCCCGAGGGGCGGGGAAAAGGGC

Table S1. DNA sequences for modified 601 nucleosome positioning sequence and synthetic oligonucleotides. 601Sb contains a modified 601 nucleosome positioning sequence (modifications are bold and underlined). Only the nontemplate strand is shown for 601Sb, AML, and HNRNPAB.
